# Nephrotoxicity of immune checkpoint inhibitors beyond tubulointerstitial nephritis: single-center experience

**DOI:** 10.1186/s40425-018-0478-8

**Published:** 2019-01-06

**Authors:** Omar Mamlouk, Umut Selamet, Shana Machado, Maen Abdelrahim, William F. Glass, Amanda Tchakarov, Lillian Gaber, Amit Lahoti, Biruh Workeneh, Sheldon Chen, Jamie Lin, Noha Abdel-Wahab, Jean Tayar, Huifang Lu, Maria Suarez-Almazor, Nizar Tannir, Cassian Yee, Adi Diab, Ala Abudayyeh

**Affiliations:** 1 Department of Nephrology, McGovern Medical School, The University of Texas Health Science Center at Houston, Houston, TX USA; 2 Division of Nephrology, Department of Medicine, David Geffen School of Medicine, University of California, Los Angeles, Los Angeles, CA USA; 3 Institute for Academic Medicine and Weill Cornell Medical College, Houston Methodist Cancer Center, Houston, TX USA; 4 Department of Pathology, McGovern Medical School, The University of Texas Health Science Center at Houston, Houston, TX USA; 5 Department of Pathology, Houston Methodist Hospital, Houston, TX USA; 6 Division of Internal Medicine, Section of Nephrology, The University of Texas MD Anderson Cancer Center, 1515 Holcombe Blvd., Unit 1468, Houston, TX 77030 USA; 7 Rheumatology and Rehabilitation Department, Assiut University Hospitals, Faculty of Medicine, Assiut, Egypt; 8 Department of General Internal Medicine, Section of Rheumatology and Clinical Immunology, The University of Texas MD Anderson Cancer Center, Houston, TX USA; 9 Department of Genitourinary Medical Oncology, The University of Texas MD Anderson Cancer Center, Houston, TX USA; 10 Department of Melanoma, The University of Texas MD Anderson Cancer Center, Houston, TX USA

**Keywords:** Checkpoint inhibitors, Immunotherapy, Glomerulonephritis, Acute tubulointerstitial nephritis

## Abstract

**Rationale & Objective:**

The approved therapeutic indication for immune checkpoint inhibitors (CPIs) are rapidly expanding including treatment in the adjuvant setting, the immune related toxicities associated with CPI can limit the efficacy of these agents. The literature on the nephrotoxicity of CPI is limited. Here, we present cases of biopsy proven acute tubulointerstitial nephritis (ATIN) and glomerulonephritis (GN) induced by CPIs and discuss potential mechanisms of these adverse effects.

**Study design, setting, & participants:**

We retrospectively reviewed all cancer patients from 2008 to 2018 who were treated with a CPI and subsequently underwent a kidney biopsy at The University of Texas MD Anderson Cancer Center.

**Results:**

We identified 16 cases diagnosed with advanced solid or hematologic malignancy; 12 patients were male, and the median age was 64 (range 38 to 77 years). The median time to developing acute kidney injury (AKI) from starting CPIs was 14 weeks (range 6–56 weeks). The average time from AKI diagnosis to obtaining renal biopsy was 16 days (range from 1 to 46 days). Fifteen cases occurred post anti-PD-1based therapy. ATIN was the most common pathologic finding on biopsy (14 of 16) and presented in almost all cases as either the major microscopic finding or as a mild form of interstitial inflammation in association with other glomerular pathologies (pauci-immune glomerulonephritis, membranous glomerulonephritis, C3 glomerulonephritis, immunoglobulin A (IgA) nephropathy, or amyloid A (AA) amyloidosis). CPIs were discontinued in 15 out of 16 cases. Steroids and further immunosuppression were used in most cases as indicated for treatment of ATIN and glomerulonephritis (14 of 16), with the majority achieving complete to partial renal recovery.

**Conclusions:**

Our data demonstrate that CPI related AKI occurs relatively late after CPI therapy. Our biopsy data demonstrate that ATIN is the most common pathological finding; however it can frequently co-occur with other glomerular pathologies, which may require immune suppressive therapy beyond corticosteroids. In the lack of predictive blood or urine biomarker, we recommend obtaining kidney biopsy for CPI related AKI.

## Introduction

Immune checkpoint inhibition had a major clinical success in clinical oncology and impacted the treatment paradigm in many cancers. The approval indications for CPI has been progressively expanding including treatments the adjuvant setting [1–3]. Immune related adverse events (irAEs) are well described toxicities that are closely associated with CPI therapies and can involve any organ in the human body [4].

Renal toxicity associated with CPI incidence has been reported as low as 2% when nivolumab alone to 4.5% when combination nivolumab and ipilimumab has been used [2, 5–7]. Guidelines for the multidisciplinary management of irAEs have been published by the Society for Immunotherapy of Cancer (SITC) and the American Society of Clinical Oncology (ASCO) [4, 7, 8]; however, the data on renal management is limited and not consistent.

The CPI-related renal pathologies are varied. Besides acute tubulointerstitial nephritis, seven other biopsy-proven kidney manifestations were published as case reports of nine patients on CPIs, including lupus nephropathy, thrombotic microangiopathy (TMA),nephrotic syndrome (focal segmental glomerulosclerosis (FSGS), two cases of minimal-change disease (MCD) [9], membranous nephropathy), pauci-immune glomerulonephritis [10], and two cases of IgA nephropathy [11–16]. The etiology of the reported kidney toxicity is not yet clear. Suggested mechanisms include direct lymphocytic cellular infiltration of renal interstitium, immune complex-mediated kidney injury, lupus nephritis, IgA, microangiopathic hemolytic anemia (TMA), or release of cytokines leading to podocyte foot process effacement (minimal-change disease and focal segmental glomerulosclerosis).

The current recommendations for the diagnosis and management of renal irAEs are not comprehensive due to the limited available data and understanding of the pathophysiology associated with renal irAEs. Therefore, we have reviewed a series of kidney biopsy cases of patients on CPIs in our institution to better understand the spectrum of injuries associated with irAEs and the treatments that were used.

## Methods

This retrospective study was approved by the institutional review board in accordance with the Declaration of Helsinki. We retrospectively identified a total of 6412 patients who had received FDA approved CPI’s at The University of Texas MD Anderson Cancer Center during 2008–2018. In addition, we collected all kidney biopsies (266) performed at MD Anderson in the same period. Of the 6412 patients, 15 patients were biopsied for suspected CPI-induced nephrotoxicity. However, an additional case in our biopsied database population that was treated with a non-approved CTLA-4 inhibitor (tremelimumab) was also identified, bringing the total cases to 16.

We collected age, sex, race/ethnicity, cancer diagnosis, name and class of CPI used, reason for kidney biopsy, underlying comorbidities including autoimmune disease, potentially nephrotoxic medications, serum creatinine at baseline, peak serum creatinine during AKI, date of last follow-up, urine sediment, proteinuria, other irAEs, serologic findings, and kidney biopsy findings.

We defined AKI using the AKIN criteria since it was used to define and categorize the severity of nephritis in the ASCO practice guidelines [17].

Patients’ renal functions were followed up for at least 3 months post-AKI before categorizing renal recovery into persistent acute kidney injury, complete renal recovery and partial renal recovery, after creatinine had reached a stable value. Complete recovery of renal function was defined by an improvement in creatinine level post-AKI to a level less than 0.35 mg/dL above the baseline. Partial recovery was defined by the serum creatinine improving to a level between the baseline plus 0.35 mg/dL and less than two times the baseline value. [18]

Complete remission in membranous nephropathy was defined by the random urine protein-to-creatinine ratios < 0.2 g/g on at least three occasions along with a normal serum creatinine. [19]

## Results

### Patient characteristics

Sixteen patients developed AKI while on CPIs and required renal biopsies over the past 10 years at our institution. The characteristics of these patients, urine findings, AKI category, and associated renal pathology are summarized in Table 1.

**Table 1:** Characteristics of the patients who developed CPI-related renal manifestations and their laboratory and microscopic findings associated with the CPI-related renal manifestations, initial therapies and the outcomes

No	Age, years	Sex	Race	Cancer type	CPI duration	Comorbidities	Potentially nephrotoxic home medication (dose; mg/day)	Baseline Cr mg/dL Prior UA	Peak Cr mg/dL Severity of AKI	Urine Sediment Cells/HPF Proteinuria	Kidney biopsy	Initial Management	Renal outcome	PFS Cancer status
Acute tubulointerstitial nephritis														
1	65	M	W	Smoldering myeloma	Pembrolizumab 6 cycles (14 weeks)	HTN, dyslipidemia, RA, GERD	Losartan, 50 Omeprazole, 20	0.8 N/A	4.83 G3	3 WBC, 1 RBC, UPC:1	• Acute TIN with eosinophils • Acute mild tubular epithelial injury with tubulitis • 5% IFTA	CPI discontinued Dexamethasone (0.6 mg/kg)	Partial recovery	17 weeks progressed to MM, started on CYBORD
2	74	M	W	Urothelial bladder cancer	Nivolumab 60 cycles (24 weeks)	CKD stage 4, stable, attributed to prior chemotherapy-related nephrotoxicity	Ibuprofen, PRN	2.5 N/A	7.48 G3	11 WBC, eosinophil 0 RBC, UPC: 0.8	• Acute TIN with neutrophils and eosinophils • Moderate hypertensive nephroscleosis • No immune complex deposition • 48% global glomerular sclerosis • 50% IFTA	CPI discontinued Prednisone (1 mg/kg)	Partial recovery followed by AKI(sepsis) dialysis-dependent	32 months Minimal residual disease
3	68	M	W	Metastatic melanoma	Nivolumab and dabrafenib and trametinib 9 cycles (9 months)	HTN, CKD stage 2, hypophysitis; hypothyroidism and adrenal insufficiency	Fosinopril, 40 Hydralazine, 30 Hydrocortisone, 60	1.3 N/A	5.38 G3	48 WBC, 7 RBC, UPC:0.36	• Acute tubuloepithelial injury • Acute tubulointerstitial nephritis • Arterial and arteriolar sclerosis • IFTA 30% and global sclerosis 23%	CPI discontinued Methylprednisolone (1.1 mg/kg) Infliximab (2 doses 8 weeks apart)	Partial recovery	15 months with no evidence of progression under observation
4	77	M	W	Papillary urothelial cancer of urinary bladder	Pembrolizumab for 10 weeks 3 doses	DM CKD stage 3 Obstructive uropathy (S/p left nephrostomy)	-	1.5 Protein (+ 1)	7.8 G4	> 182 WBC 9 RBC eosinophil + 1 protein	ATIN with eosinophil and few multinucleated giant cells ATN Global sclersosis 50% and IFTA 50%	CPI discontinued. Methyprednisone 1 mg/kg BID intiated on HD and steroid dose was tapered	Persistent AKI dialysis depenedent	2 months with no evidence of progression under observatoin
5	55	M	B	Transitional cell bladder cancer	Atezolizumab around 6 months	Obstructive uropathy s/p bilateral nephrostomy tubes CKD stage 4 GERD	Pantoprazole, 40	3.3 UPC 1.2	5.8 G3	27 WBC 8 RBC eosinophil UPC:2.7	Acute and chronic tubulointerstitial nephritis with neutrophils and eosinophils Diffuse (> 95%) IFTA	CPI discontinued.	no renal recovery. CKD stage 5	9 months had progression of metastasis. Deceased
Acute tubulointersitial Nephritis with Glomerulonephritis														
6	41	M	W	Squamous cell cancer of the lung	Nivolumab 4 cycles (14 weeks)	Asthma	Ibuprofen daily for 2 weeks	0.8 N/A	4.52 G3	19 WBC, 320 RBC,UACR:1025 mg/g	• Acute focal segmental necrotizing pauci-immune GN (no crescents or global sclerosis): ANCA-negative • Mild interstitial nephritis without atrophy	CPI discontinued Prednisone (1 mg/kg) Rituximab (1 dose)	Complete recovery	14 weeks patient deceased owe to progression of cancer
7	75	M	W	Metastatic RCC	Tremelimumab 2 doses (6 weeks)	HTN and CKD stage 3	Amoxicillin/clavulanate, 500 mg daily for 5 days Hydralazine, 75	1.8 N/A	4.75 G3	5 WBC, 67 RBC,UPC:1.43	• Acute focal segmental pauci-immune necrotizing GN • Mild acute tubulointerestitial nephritis with eosinophils • Acute tubular epithelial injury • Arterial and arteriolar sclerosis • IFTA 5% and global sclerosis 38%	CPI discontinued Methylprednisolone (2 mg/kg) Rituximab (weekly for 4 doses) Plasmapheresis (daily for 5 sessions)	Partial recovery	11 months with no evidence of progression under observatoin
8	69	W	W	Uveal Melanoma	Nivolumab and Ipilimumab (3 cycles) 9 weeks	HTN, DM, Stroke CKD stage 3 GERD	Omeprazole, 40 Valsartan, 80	1.4 No protein	4.9 G3	15 WBC 7 RBC UPC:0.4	Granulomatous necrotizing vasculitis hypertensive nephrosclerosis Patchy moderate to severe interstitial inflammation 50% global glomeulosclerosis and 30% IFTA Negative ANCA	CPI discontinued. Prednisone 1 mg/kg daily followed by rituximab x1 after one week	Complete recovery	8 months with no evidence of progression under observatoin
9	69	M	W	Melanoma	Ipilimumab and Nivolumab 2 cycles (6 weeks)	GERD, HTN, CKD stage 3	Olmesartan, 40 Furosemide, 20 Omeprazole, 20	1.4 N/A	2.40 G2	7 WBC, 11 RBC, UPC: 7.7	• IgA nephropathy with focal segmental endocapillary hypercellularity and sclerosis • Acute mild TIN with eosinophils • 40% global glomerular sclerosis, 20% IFTA • Mild arterial and arteriolar sclerosis	CPI discontinued Prednisone (0.5 mg/kg)	Complete recovery followed by relapse	19 months with no evidence of disease on observation
10	50	F	W	Melanoma	Pembrolizumab completed 5 doses (12 weeks)	Asthma, GERD, HTN	Naproxen, 250 PRN Omeprazole, 10 HCTZ, 12.5	0.8 N/A	3.08 G3	6 WBC, 2 RBC, negative dipstick	Done 5 weeks after AKI: • low-grade tubulointerstitial injury • IgA nephropathy (without pathologic indication of active disease) • FSGS, NOS • Very mild interstitial inflammation	CPI discontinued Prednisone (2 mg/kg) Mycophenolate Mofetil 1 g BID Infliximab (one dose)	Partial recovery followed by AKI attributed to Vemurafenib	4 weeks progression of metastasis
11	60	F	H	RCC	Nivolumab 6 cycles (16 weeks)	GERD, and dyslipidemia	Esomeprazole, 40	0.8 Negative dipstick	N/A	2 WBC, 3 RBC, UPC: 9.7	• PLA2R negative early membranous GN • Focal T-cell–rich crescent-like inflammation • Acute tubulocentric TIN with T cells positive for CD3, CD4, CD8	CPI discontinued Prednisone (1 mg/kg)	Complete recovery	20 weeks then had disease progression started on axitinib
12	61	F	W	Smoldering myeloma	Pembrolizumab 2 cycles (8 weeks)	Hypothyroidism,HTN, dyslipidemia GERD	Lansoprazole, 30	0.6 N/A	2.86 G3	32 WBC, 1 RBC, UPC: 0.3	• Granulomatous TIN • C3 deposition (possible early GN) • Rare subepithelial deposits • 5–10% IFTA • Arterial and arteriolar sclerosis	CPI discontinued Prednisone (1 mg/kg)	Partial recovery	12 months with no progression under observation
13	74	M	W	RCC CML	Nivolumab with Axitinib (for 14 months) and Imatinib (for 20 months)	HTN CKD stage 3 GERD	Omeprazole, 40	1.6 N/A	2.73 G2	1 WBC, 0 RBC, UPC: 0.38	• Acute tubuloepithelial injury • Acute tubulointerestitial nephritis with eosinophils • FSGS (preservation of foot process) likely secondary (HTN and post-nephrectomy) • Arterial and arteriolar sclerosis (moderate) • IFTA 20% and global sclerosis 9%	CPI discontinued Predisone (0.8 mg/kg)	Partial recovery	12 months with evidence of progression
14	63	M	W	Chondroma	Pembrolizumab 6 cycles (18 weeks)	Coronary artery disease, hypothyroidism, neurogenic bladder	–	0.5 N/A	2.25 G3	21 WBC, 11 RBC, UPC: 31	• AA type amyloidosis, • Acute tubular epithelial injury • 28%global glomerular sclerosis • 5% IFTA	CPI discontinued Methylprednisolone (1 mg/kg) Infliximab 440 mg one dose	Partial recovery followed by AKI(sepsis)	26 weeks Patient deceased owing to bowel perforation
Cases with suspected CPI toxicity														
15	38	M	W	Hodgkin Lymphoma	Nivolumab and LAG-3 antibody 2 cycles (10 weeks)	Cardiomyopathy s/p SCT (9 months ago)	Sulfamethoxazole and trimethoprim (800/160 mg) 3 times per week Valacyclovir, 500 Pantoprazole, 40	0.8–0.9 N/A	1.63 G1	11 WBC, 1 RBC, UPC: 0.05	Done 4 weeks after AKI (first biopsy was inadequate): • No evidence of acute glomerular or tubular injury or inflammation • IFTA 5% and global sclerosis 5%	CPI was held then resumed after 6 weeks along with proton pump inhibitor without recurrence of AKI	Complete recovery	13 months remains with complete response then patient declined further therapy
16	58	M	W	Non-small cell lung cancer	Carboplatin and Pemetrexed for 3 cycles (7 weeks added to Pembrolizumab (13 weeks)	HTN COPD	Amoxicillin and Clavulanate, 875–125 mg BID Lisinopril 20	0.5 Protein (+ 1)	7.1 G3	No pyuria or hematuria UPC 0.6	ATN No Glomerulosclerosis 15% IFTA	CPI discontinued. Prednisone 1 mg/kg	Persistent AKI dialysis dependent depenedent	9 months with no recurrence (withdrew from further therapy)

*PFS* progression-free survival, *M* male, *F* female, *W* white, *B* black, *LAG-3* lymphocyte activation gene 3, *HTN* hypertension, *GERD* gastroesophageal reflux disease, *MM* multiple myeloma, *RA* rheumatoid arthritis, *DM* diabetes mellitus, *COPD* chronic obstructive pulmonary diseases, *SCT* stem cell transplant, *CKD* chronic kidney disease, *WBC* white blood cells, *RBC* red blood cells, *UA* urinalysis, *UPC* urine protein to creatinine ratio, *WNL* within normal limit, *ANA* anti-nuclear antibody, *ANCA* antineutrophil cytoplasmic antibody, *RF* rheumatoid factor, *CCP* cyclic citrullinated peptide, *MPO* myeloperoxidase, *CK* creatine kinase, *N/A* not available, *dsDNA* double-stranded DNA, *GN* glomerulonephritis, *TIN* tubulointerstitial nephritis, *IFTA* interstitial fibrosis/tubular atrophy, *AA* amyloid A, *UACR* urine albumin to creatinine ratio, *PET* positron emission tomography, *FSGS* focal segmental glomerulosclerosis, *CPI* immune checkpoint inhibitor, *BID* twice daily, *Cr* creatinine, *RRT* renal replacement therapy

Most cases identified were white men (1 case was a Hispanic man, and 4 cases were women), with a median age of 64 years (range, 38–77 years). Renal cell carcinoma, urothelial bladder cancer and melanoma were the most common malignancies (3 cases of RCC and 3 urothelial bladder cancer and 4 cases of melanoma), followed by multiple myeloma (2 cases) and 1 case each of chondroma, squamous cell cancer of the lung, adenocarcinoma of the lung, and Hodgkin lymphoma. Most cases occurred in the setting of nivolumab (anti-PD-1) and pembrolizumab (anti-PD-1) use (6 cases each), a combination of nivolumab and ipilimumab (anti-CTLA-4) (2 cases), tremelimumab(anti-CTLA-4) (1 case), and atezolizumab (anti-PD-L1) (1 case). 7 patients had chronic kidney disease (CKD) at baseline:5 had CKD stage 3, and 2 had CKD stage 4.

### Clinical features

The median time to development of AKI after starting a CPI was 14 weeks (range: 6–56 weeks). However, AKI occurred within 9 weeks with the use of the CTLA-4 inhibitor tremelimumab or the combination of the CTLA-4 inhibitor ipilimumab and the PD-1 inhibitor nivolumab. All other patients on PD-1 inhibitors had longer durations to development of AKI: a median of 20 weeks (range, 10–56 weeks) with nivolumab alone and 13.5 weeks (range: 8–18 weeks) with pembrolizumab alone.

The most common urine finding was sub-nephrotic proteinuria at time of acute kidney injury diagnosis (urine studies were done within 48 h of diagnosis in 13 out of 16 cases). The median urine protein-to-creatinine (UPC) ratio was 0.8 g/g with a range of 0–31. 3 cases had ≤0.3 g/g protein in the urine. 10 cases had proteinuria ranging from 0.3 to 3 g/g, and 3 cases had nephrotic-range proteinuria and hypoalbuminemia consistent with nephrotic syndrome and associated with renal pathologies of AA amyloidosis, membranous glomerulonephritis, or IgA nephropathy (one case each). Prior urinalysis was not available in most of the cases (11 out of 16) to compare the acuity of reported proteinuria. Pyuria (> 5 white blood cells [WBC]/high-power field [HPF]) with associated biopsy finding of tubulointerstitial inflammation was present in 7 patients but was absent in four patients despite histological evidence of tubulointerstitial nephritis in those patients, there was no clear association with type of CPI or use of steroids. Microscopic hematuria (> 3 red blood cells [RBC]/HPF) was present in eight of the patients in our series, two patients had > 50 RBC/HPF with a renal pathology of pauci-immune glomerulonephritis.

Among non-renal irAEs that developed during therapy with CPI (both Anti-PD-1 and Anti -CTLA-4), the most common irAE was hypothyroidism. Other irAEs were dermatitis, pnemonitis, colitis, esophagitis, adrenal insufficiency, and myositis. Majority of the non renal irAEs developed nephrotoxicity after or at the time of non renal irAE diagnosis. No correlation was observed between the severity and recovery of non-renal irAE with the renal one.

Summary of observed other irAEs in patients who developed CPI related nephrotoxcity and their outcome are included in Table 2.

**Table 2:** Observed irAEs in patients who developed CPI related nephrotoxcity and their outcome

Patient #	CPI	AKI severity	Assosciated irAE	Relation to AKI diagnosis	Renal and non renal irAE outcome
11	Nivolumab	Nephrotic syndrome	Hypothyrodisim (G2)	4 weeks prior to AKI	Persistent hypothyrodisim Complete remission of nephrotic syndrome
14	Pembrolizumab	G3	Colitis (G3)	2 weeks prior to AKI	Diarrhea and renal function improved partially then patient developed 2nd AKI
2	Nivolumab	G3	Elevated dsDNA and RNP titers	At the time of AKI diagnosis	Titers became undetectable after 4 week Partial renal recovery
6	Nivolumab	G3	Hypothyrodisim (G2)	10 weeks prior to AKI	Persistent hypothyrodisim Complete renal recovery
3	Nivolumab	G3	Myositis	6 weeks after AKI	Myosisits had resolved Partial renal recovery
7	Tremelimumab	G3	Dermatitis (G1) Pneumonitis (G2)	At the time of AKI diagnosis	Dermatitis and pneumonitis had resolved within 2 week Partial renal recovery
15	Nivolumab	G1	Hypothyrodisim (G2) Esophagitis (G2)	5 weeks prior to AKI	Esophagitis and AKI had fully recovered Persistent hypothyrodisim
10	Pembrolizumab	G3	Dermatitis (G1) Pneumonitis (G2)	At the time of AKI diagnosis	Dermatitis and pneumonitis had resolved within 1 week Partial renal recovery
8	Nivolumab and Ipilimumab	G3	Dermatitis (G1) Thyroditis (G3) Adrenal insuffiency (G1)	5 weeks prior to AKI	Persistent hypothyrodisim and adrenal insuffiency Complete renal recovery

Common Terminology Criteria for Adverse Events (CTCAE)

Grade 1 Mild; asymptomatic or mild symptoms; clinical or diagnostic observations only; intervention not indicated

Grade 2 Moderate; minimal, local or noninvasive intervention indicated; limiting ageappropriate instrumental ADL

Grade 3 Severe or medically significant but not immediately life-threatening; hospitalization or prolongation of hospitalization indicated; disabling; limiting self care ADL

Grade 4 Life-threatening consequences; urgent intervention indicated

*CPI* immue Checkpoint inhibitor, *AKI* acute kidney injury, *irAE* immue related adverse events

### Renal pathologies and their associated clinical findings

#### Acute tubulointerstitial nephritis

Tubulointerstitial inflammation was the most common pathologic finding on biopsy, present in 14 of the 16 cases as either the main microscopic finding or as a mild form of interstitial inflammation in association with other glomerular pathologies.

As presented classically in the literature, our cases include 5 cases with only ATIN of which all had eosinophilic infiltration in addition to neutrophils except for one case. 2 cases were treated with pembrolizumab, 2 cases on Nivolumab, and 1 case was treated with Atezolizumab. 3 out of the 5 cases were also treated with either Ibuprofen or proton-pump inhibitors prior to CPI use which are also associated with ATIN [20].

#### Acute tubulointersitial Nephritis & Glomerulonephritis

##### Pauci-immune glomerulonephritis (Fig. 1)

Acute focal segmental necrotizing pauci -immune glomerulonephritis was noted in 3 cases, with nivolumab in 1 case, tremelimumab in 1 case, and nivolumab combined with ipilimumab, in 1 case. The patient on nivolumab had non-specific symptoms of fatigue and generalized weakness. Antineutrophil cytoplasmic antibody (ANCA) titer was negative.

**Fig. 1:**
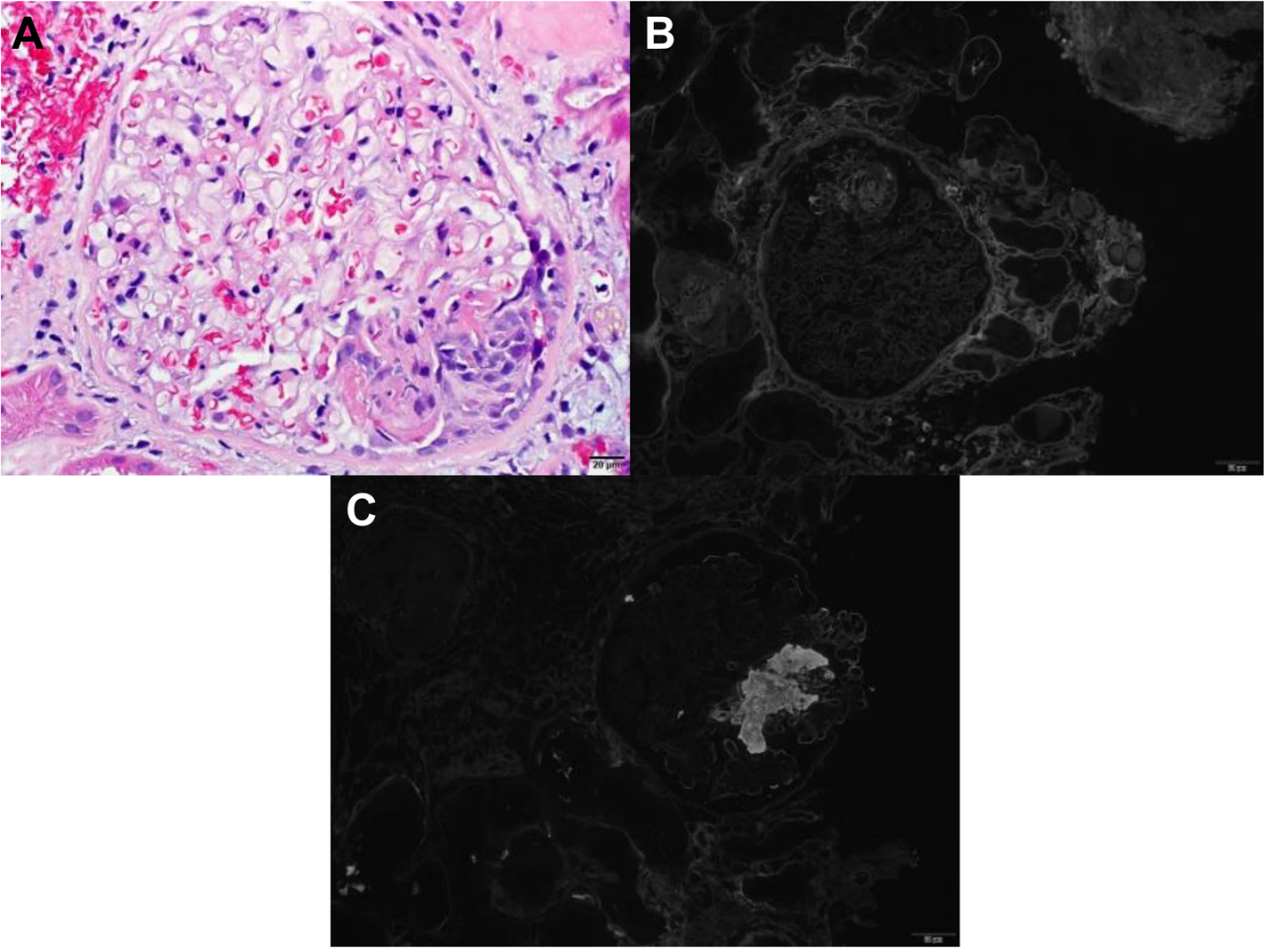
Two cases showed pauci-immune glomerulonephritis characterized by focal, segmental glomerulonecrosis (**a**, H&E) without immune complex deposition (**b**, IgG immunofluorescence) and with fibrin deposition within the lesions (**c**, fibrinogen immunofluorescence)

The patient who developed pauci-immune glomerulonephritis related to tremelimumab had arthralgia, vasculitic rash, and pneumonitis. Serologic findings were remarkable for positive antinuclear antibodies (1:160), positive myeloperoxidase-antineutrophil cytoplasmic antibodies (*MPO*-*ANCA; level > 8*), negative anti-glomerular basement membrane (anti-GBM) antibodies, and normal complement.

The 3rd case we observed with microscopic finding of pauci-immune granulomatous necrotizing vasculitis occurred in a patient treated nivolumab combined with ipilimumab. The patient had AKI with nonspecific symptoms of poor appetite and fatigue. ANCA and anti-GBM titers were negative. Fungal stains and stains for acid-fast bacilli and BK polyomavirus were negative which are also associated with granulomatous tubulointerstitial inflammation. In all 3 vasculitis cases urine studies indicated microscopic hematuria, pyuria and sub nephrotic proteinuria were seen.

##### IgA nephropathy (Fig. 2)

IgA nephropathy developed in a patient receiving the combination therapy of ipilimumab and nivolumab and in a patient receiving pembrolizumab. Both patients had hypertension with no prior history of IgA nephropathy. No previous urine studies were available for evaluation of prior reported microscopic hematuria or proteinuria. The patient who was receiving ipilimumab combined with nivolumab had preexisting stable CKD stage 3 (eGFR 55–60 mL/min/1.73m^2^) and had an increase in creatinine level from a baseline of 1.3 mg/dL to 2.4 mg/dL, with pyuria and hematuria (7 and 11 cells/HPF respectively) and nephrotic range proteinuria (UPC ratio: 7.7 g/g) after completing the 2nd cycle. Kidney biopsy showed IgA nephropathy with focal, segmental endocapillary hypercellularity and sclerosis and mild ATIN with eosinophils.

**Fig. 2:**
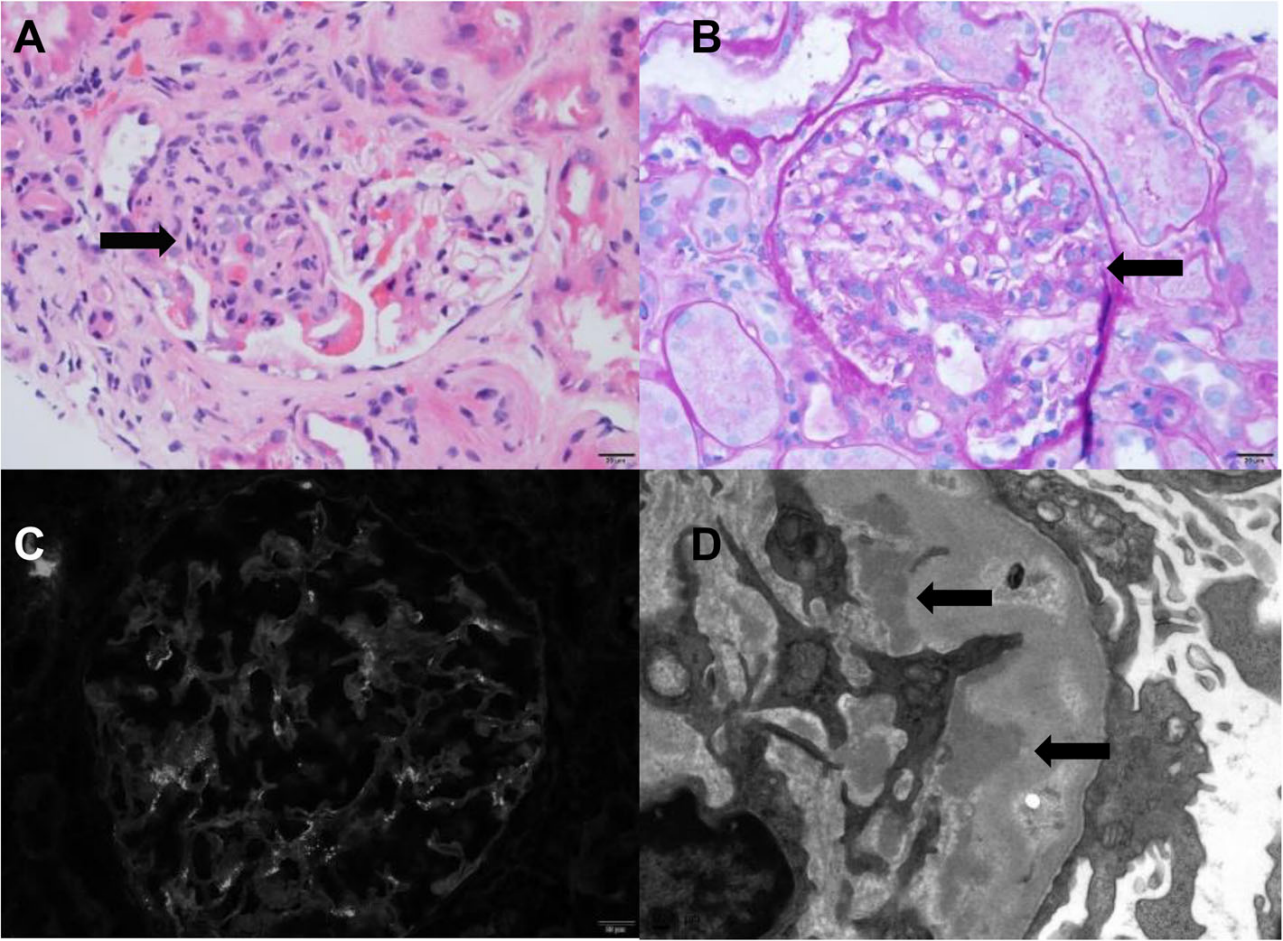
Two cases showed IgA nephropathy, one of which was characterized by segmental mesangial and endocapillary hypercellularity seen on H&E (**a**) and PAS stains (**b**), indicated by arrows. There were IgA-dominant immune complex deposits (**c**, IgA immunofluorescence) with numerous mesangial electron dense deposits ultra-structurally, indicated by arrows (**d**, electron microscopy).

The 2nd case developed AKI after receiving the 5th cycle of pembrolizumab. Urinalysis was positive for pyuria with no hematuria or proteinuria. Biopsy showed IgA nephropathy with focal, segmentally sclerotic glomeruli, without evidence of active disease along with mild ATIN. Of note, on the second case, the biopsy was performed 5 weeks after discontinuation of the CPIs and while the patient was on prednisone, which may account for the lack of significant inflammation in the biopsy.

##### Membranous nephropathy (Fig. 3)

One patient who had had negative urinalysis results before starting nivolumab developed nephrotic-range proteinuria after 6 cycles (16 weeks of therapy) with a UPC ratio of 9.7 g/g and no hematuria, pyuria, or significant change in eGFR. The pathology showed features of early membranous glomerulonephritis negative for anti-phospholipase -A2-receptor (PLA2R) autoantibodies with focal T cell–rich crescent-like inflammation and acute glomerulocentricnephritis with T cells positive for CD3, CD4, and CD8. The presence of concurrent ATIN, and the patient’s complete recovery (UPC ratio improved to < 0.5 g/g) after CPI discontinuation and steroid therapy suggested that the membranous nephropathy was related to CPI rather than to progression of the underlying malignancy. The rest of the secondary serologic analysis, including the hepatitis panel, was negative.

**Fig. 3:**
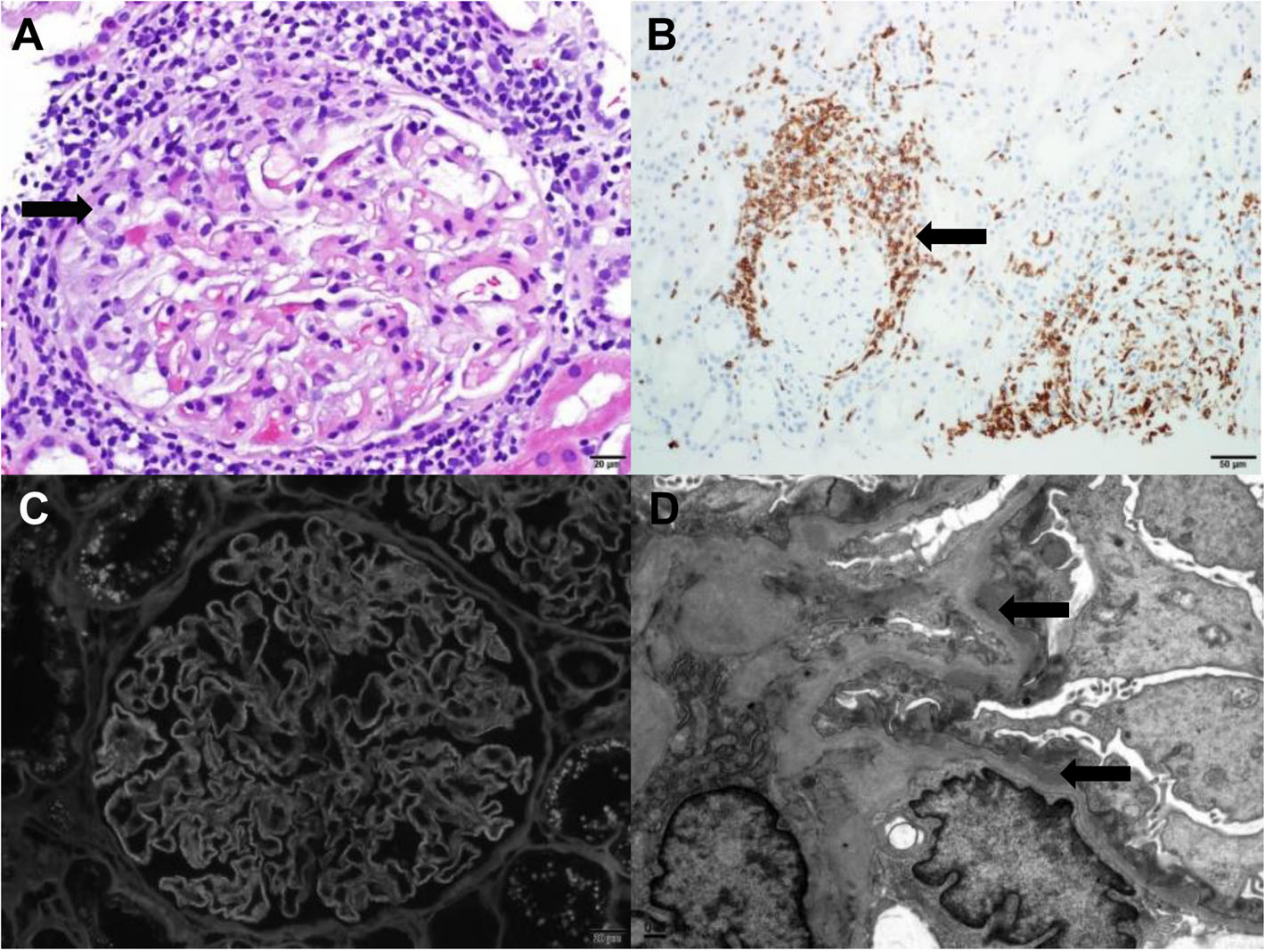
A single case of membranous glomerulonephritis which showed a focal area of crescent-like inflammation and glomerulocentric tubulointerstitial nephritis (**a**, H&E, arrow indicates crescent like inflammation) which was T-cell rich (**b**, CD3 immunohistochemistry, arrow indicated CD3 positive cells). There was diffuse capillary immune complex deposition (**c**, IgG immunofluorescence) with numerous subepithelial electron-dense deposits, indicated by arrows (**d**, electron microscopy)

##### C3 glomerulopathy

One patient with smoldering multiple myeloma was treated with pembrolizumab for 2 cycles and then developed AKI with microscopic hematuria and pyuria. The patient had granulomatous tubulointerstitial nephritis. Glomeruli were normal in appearance without inflammation. However, there was granular C3-*only* deposition on immunofluorescence with corresponding rare, large sub-epithelial deposits by electron microscopy and normal serum C3 levels. Lack of immunoglobulin and light chain deposition was confirmed by immunofluorescence staining of proteinase-treated paraffin sections. The patient had no evidence of infection and had stable free kappa light chains. These findings suggested early features of C3 glomerulopathy.

##### Focal segmental glomerulosclerosis (FSGS)

FSGS was observed in the kidney biopsy of one case treated with nivolumab as monotherapy. The patient had hypertension and proteinuria, but it was less than 0.5 g per day (UPC ratio < 0.5 g/g) with no hematuria or significant pyuria noted on the urinalysis. Electron microscopy showed preservation of foot processes which is suggestive of secondary focal segmental glomerulosclerosis. Again, acute tubulointerstitial nephritis was noted with eosinophilia.

##### AA amyloidosis (Fig. 4)

After 18 weeks (6 cycles) of pembrolizumab, a patient with chondroma developed AKI with nephrotic-range proteinuria (UPC ratio: 31 g/g) and severe colitis. The kidney biopsy was positive for amyloid on Congo red and thioflavin T. Immunofluorescence was negative for light chains, and mass spectroscopy confirmed AA amyloid. Patient was started on steroids 48 h prior to the biopsy and could explain the lack of ATIN in the pathology although there was evidence of microscopic hematuria and pyuria on the urinalysis.

**Fig. 4:**
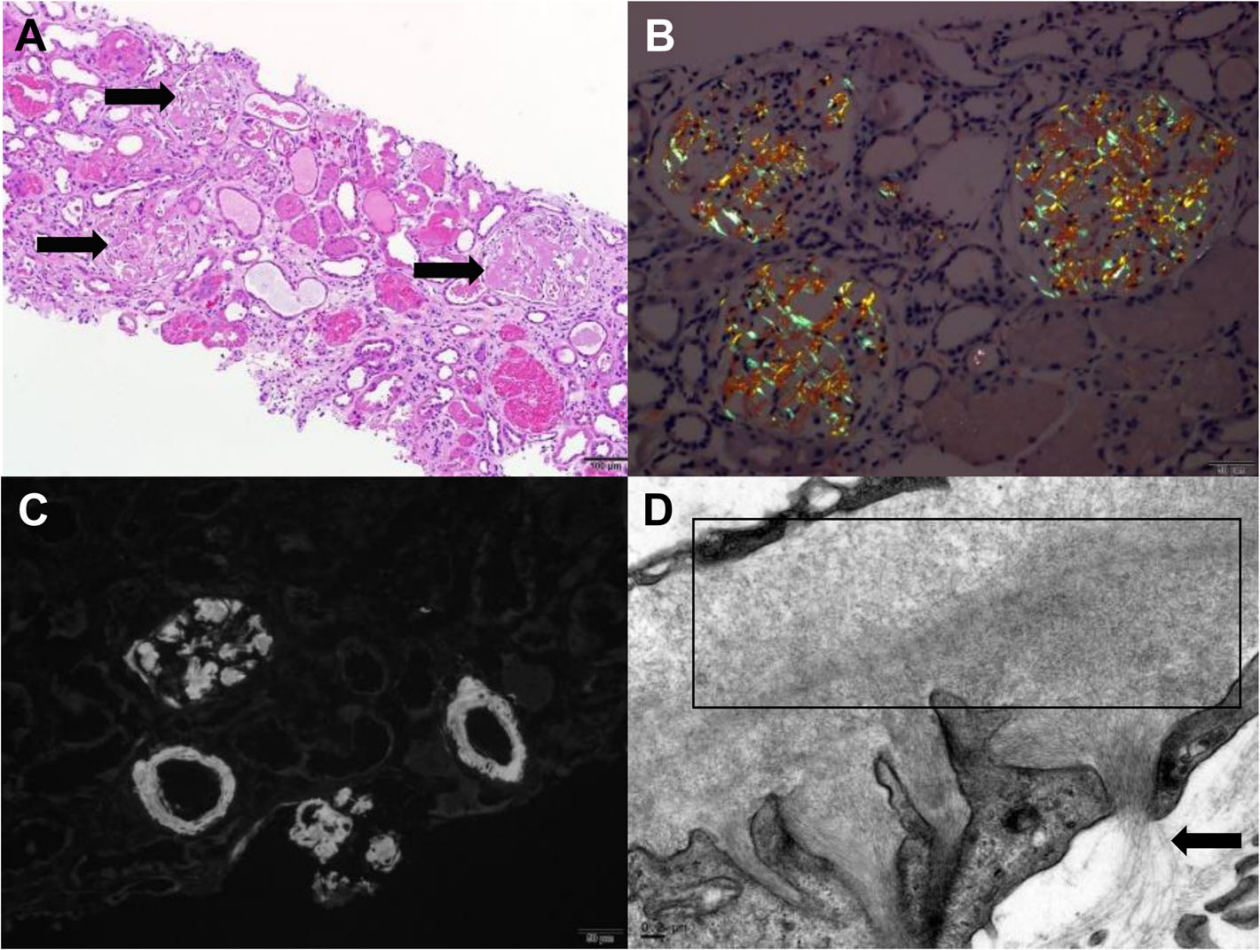
In the case of amyloidosis, the glomeruli showed diffuse mesangial expansion by amorphous eosinophilic matrix, indicated by arrows (**a**, H&E) that showed green birefringence under polarized light by Congo red stain (**b**) and diffuse 3+ fluorescence (Thioflavin T stain) of deposits in glomeruli and arterioles (**c**). Abundant fibrils measuring 8–10 nm were identified by electron microscopy, area of dense fibril deposition within the box, less dense fibril deposition indicated by arrows (**d**)

Comparison of the characteristics and renal outcomes of patients with renal pathologies related to CPI use between the current study and the previously published case reports are summarized in Table 3.

**Table 3:** Comparison of the characteristics and renal outcomes of patients with CPI related nephropathy between the current study and the previously published case reports

Case	Renal Manifestation	Urine studies/ Serology	Malignancy	Immunotherapy	Therapy	Response
Nephrotic syndrome cases in relation to immune checkpoint agents						
Daanen et al. [13]	FSGS	–	RCC	Nivolumab	D/C + steroids+ MMF	Remission followed by relapse
Kitchlu et al. [14]	MCD	–	Hodgkin lymphoma	Pembrolizumab	D/C + steroids	Remission (partial)
Kitchlu et al. [14]	MCD	–	Melanoma	Ipilimumab	D/C + steroids	Remission
Lin et al. [9]	Membranous Nephropathy (PLA2R neg.)	–	Melanoma	Nivolumab	D/C + steroids	Remission (partial)
Current study (#11)	Membranous Nephropathy (PLA2R neg.)	–	RCC	Nivolumab	D/C + steroids	Remission
IgA nephropathy cases in relation to immune checkpoint agents						
Jung et al. [16]	AKI grade 4 Cellular crescents with necrosis Sub-epithelial desposition.	Proteinuria and hematuria	Clear cell Kidney cancer	Nivolumab	D/C, steroids and RRT	Recovery (RRT was d/c after 5 months)
Kishi et al. [15]	AKI grade 2 Mesangial exp. with no crescents or endocapillary hypercellularity	Sub nephrotic proteinuria. Hematuria	Lung SCC	Nivolumab	D/C	Remission (Complete)
Current study (#9)	AKI grade 2 endocapillary hypercellularity	Nephrotic range proteinuria Pyuria and hematuria	Melanoma	Nivolumab+ Ipilimumab	D/C and steroids	Remission followed by relapse
Current study (#10)	AKI grade 3 No Glomerular proliferative lesions*	No proteinuria No hematuria +pyuria	Melanoma	Pembrolizumab	D/C and steroids, MMF, and infliximab	Partial recovery
Pauci-immune GN cases in relation to immune checkpoint agents						
Van den Brom et al. [12]	GPA ** Dysmorphic erythrocytes and proteinuria Extra renal: Cutaneous vasculitis Stable lung nodule	+PR3-ANCA C; normal	Malignant Melanoma	Ipilimumab followed by Pembrolizumab	Cyclosporine and steroids	Remission
Cusnir et al. [10]	GPA Focal proliferative GN Extra renal; Cutaneous vasculitis sinusitis	+PR3-ANCA C; N/A	Malignant Melanoma	Nivolumab+ Ipilimumab	steroids and rituximab	Not Stated
Current study (#6)	Focal necrotizing pauci-immune glomerulonephritis with no crescents Extra renal; N/A	Negative ANCA C; N/A G3	NSCLC (SCC)	Nivolumab	D/C, steroids and rituximab	Complete recovery
Current study (#7)	Focal segmental pauci-immune necrotizing glomerulonephritis Extra renal; N/A	+MPO-ANCA C; normal G3	mRCC	Tremelimumab	D/C, steroids, plasmaphresis and rituximab	Partial recovery
Current study (#8)	Granulomatous necrotizing vasculitis Extra renal; N/A	Negative ANCA C3/4 normal	Uveal Melanoma	Nivolumab+ Ipilimumab	D/C, steroids and rituximab	Complete recovery
Anti-dsDNA cases in relation to immune checkpoint agents						
Fadel et al. [11]	AKI with proteinuria Extramembranous and mesangial deposits (IgG, IgM, C3, C1q)	+dsDNA C; normal	Metastatic Melanoma	Ipilimumab	D/C	Partial renal recovery dsNDA; not detectable
Current study (#2)	AKI with proteinuria ATIN with no I.C. deposition GN	+dsDNA and RNP	Bladder cancer	Nivolumab	D/C and steroids	Partial renal recovery dsNDA and RNP; not detectable

*FSGS* focal segemental glomerulosclerosis, *MCD* mininmal change disease, *D/C* immune checkpoint agent was discontinued, *Neg* Negative, *PLA2R* anti-phospholipase-A2 receptor, *AKI* acute kidney injury, *I.C* immune complex, *GN* glomerulonephritis, *C*, complement, *Exp.* expansion, *AKI* acute kidney injury, *ATIN* acute tubulointerstitial nephritis, *RRT* renal replacement therapy, *GPA* granulomatosis with polyangiitis, *PR3* proteinase 3, *ANCA* antineutrophil cytoplasmic antibodies, *MPO* myeloperoxidase, *N/A* not available, *NSCLC* non-small cell lung cancer, *mRCC* metastatic renal cell carcinoma, *dsDNA* double stranded DNA

*Rena**l** biospy was done 5 weeks post treatment with steroid, MMF and infliximab

**Presumptive diagnosis. Renal Biopsy was not reported

### Treatment

CPIs were discontinued in 15 cases and held for 6 weeks in one case of the studied cases at the time of AKI diagnosis. Most of the patients received steroids at the time of AKI diagnosis except two patients. One who had mild nephritis, in whom CPI was held, and another who had severe kidney disease and poor residual renal function (seen in interstitial fibrosis with tubular atrophy (IFTA) > 90% on biopsy). Additional immunosuppressant agents were used as indicated by the associated glomerulonephritis in the pathology. The dose, form, or duration of steroid treatment did not follow any guideline. The initial used dose of prednisone ranged from 0.5–4 mg/kg/day. The prednisone was tapered off over 4–24 weeks depending on the renal pathology and recurrence of renal disease.

### Renal function and survival outcomes

Three of the 5 ATIN cases all had partial renal recovery after prednisone and one of which had also infiliximab (2 doses). The 2 cases with no renal response, one had poor residual renal function from severe chronic kidney disease with associated IFTA > 90%, CPI was held, and no steroids were given, and the other case had associated acute tubular necrosis and remained hemodialysis dependent as of 12 weeks post AKI diagnosis despite prednisone therapy.

In our glomerulonephritis cases, membranous nephropathy, granulomatous with C3 glomerular deposition, and focal segmental glomerulosclerosis, and one of the 2 cases of CPI-related IgA nephropathy had complete or partial recovery after starting prednisone (0.5 mg/kg-3 mg/kg). The two ANCA negative pauci-immune glomerulonephritis patients had complete recovery of renal function after discontinuing CPI and starting prednisone and rituximab for treatment of pauci immune GN. The 3rd patient with ANCA positive pauci immune GN underwent treatment with steroids, plasmapheresis, and rituximab as indicated for creatinine > 4.0 mg/dl and possible lung involvement. The patient with AA amyloid had partial renal recovery after steroid treatment and due to continued colitis received infliximab and later progressed to AKI due to sepsis. The second IgA case had partial renal response after steroids, mycophenolate mofetil and then one dose of infliximab due to failure of initial response.

Three of the 16 cases died because of disease progression. All 13 surviving patients continued treatment, with 5 being on active therapy and 8 staying on surveillance.

We included the disease progression free survival (PFS) of the 16 patients in Table 1. However, we are not able to conclude a disease response or PFS benefit in these patients since we do not have in this current population patients who were treated with CPIs and didn’t develop nephrotoxicity to make such comparison.

## Discussion

We report 16 cases, 5 with typical ATIN with no associated glomerulonephritis which is the most commonly reported etiology for AKI related to CPIs [21] . However, we have also presented ATIN associated with glomerulopathies in nine out of the 16 cases. Some of these glomerulopathies have not been reported in the literature to be associated with CPI use (MPO-ANCA positive pauci-immune glomerulonephritis, C3 staining, or AA amyloid). The treatments of these glomerulopathies were varied and not limited to steroids but included other immunosuppressive medications in steroids refractory cases and as a standard of care to treat the glomerulopathies.

Immunotherapy-related acute interstitial nephritis [22, 23] could be due to the loss of tolerance of drug-specific effector T cells with the inhibition of PD-1 signaling. These are T cells that were primed during a prior nephritogenic drug exposure. Another proposed mechanism is the development of autoimmunity to kidney self-antigens after the loss of self-tolerance and potentiation of antigen recognition after blocking of the CTLA-4 or PD-1 pathway, which plays an important role in regulating peripherally and at the level of target organs, respectively [24].

The types of nephropathies induced by the CPI class vary tremendously, even when induced by a single agent such as nivolumab. For a given glomerulopathy related to CPIs, the severity and the response to steroids can also differ, partly due to patient differences. Overall, the variety of CPI-induced renal manifestations suggests multiple complex mechanisms that should be further elucidated.

Autoantibody development was believed to explain the variability in immune- related adverse effects [25, 26]. We observed one case with acute interstitial nephritis that was refractory to high-dose corticosteroids. The patient did respond partially to infliximab. This was also demonstrated in one of the IgA cases where patient was refractory to high dose steroids and Cellcept and finally responded after infliximab, suggesting that adding anti–tumor necrosis factor alpha could have mediated some of the renal inflammation. The treatment success may be due to infliximab’s immediate action to block TNF-alpha, which is usually upregulated in patients on CPIs [23]. The renal benefit could be direct or indirect, via a decrease in cytokine release that would otherwise contribute to acute tubular necrosis. However, a delayed effect of mycophenolate mofetil use, which can take several weeks, cannot be excluded in one of our cases [27]. Adding infliximab as a second-line therapy for cases with irAEs that are resistant to steroids has been suggested previously [27, 28], but has not specifically reported in previous cases of nephritis-type irAEs.

Based on the variety of renal pathologies noted in our 16 patients who developed AKI while on CPIs, we recommend a kidney biopsy in patients with grade 2 or above renal injury and/or patients with unexplained proteinuria of greater than one gram/day. At baseline, the urinalysis, spot protein to creatinine ratio, antinuclear antibody levels, and anti-double-stranded DNA levels could be obtained before initiation of the CPI therapy.

### Limitations

Patients with other irAEs may have also undergone treatment with steroids or other forms of immunosuppression that further ameliorate the renal dysfunction prior to the renal biopsy. In addition, because of the retrospective nature of the study we do lack baseline urinalysis prior to use of CPI and therefore cannot completely exclude the presence of underlying renal pathologies prior to CPI use. In addition, although majority of our cases with renal pathologies were treated with anti-PD-1 agents we cannot conclude that ATIN was more prevalent in this class of medications since out of the 6412 patients identified more than half (3608) were treated with anti-PD1 agents. Needed are prospective studies with urinalysis, proteinuria evaluation at baseline, during CPI and pre-toxicity, at time of toxicity and at the time of toxicity resolution to more accurately identify and characterize renal irAEs and response to immune suppression, in addition to obtaining early renal consult and renal biopsy when indicated.
